# Supplementary material for: Deletion of the Serotonin Receptor 7 Gene Changed the Development and Behavior of the Mosquito, Aedes aegypti
Source: Insects. 2022 Jul 25;13(8):671. doi: 10.3390/insects13080671 (PMC9332693; doi:10.3390/insects13080671)
Supplement: Supplementary file 1 [file insects-13-00671-s001.zip › insects-1793699-supplementary.pdf]

Supplementary Information

Supplementary Figures

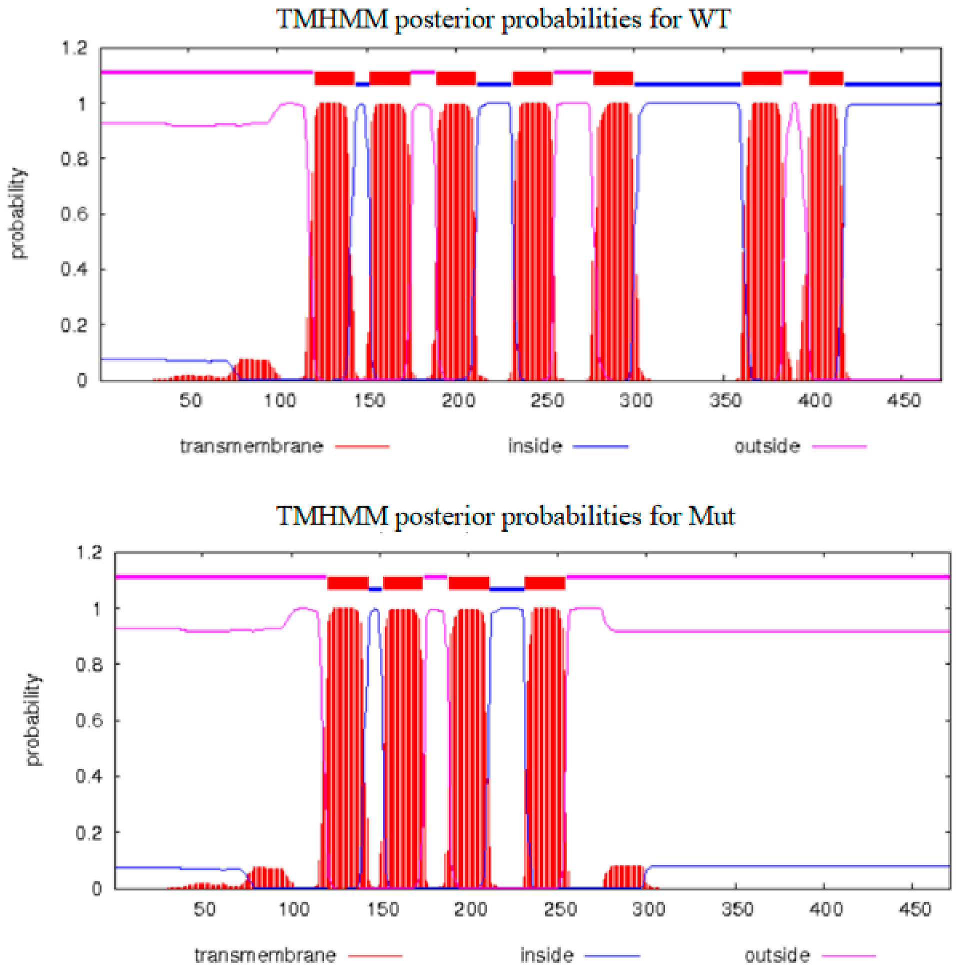

Figure S1. The WT and mutant 5-HTR7A transmembrane domains. TMHMM 2.0 online software (TMHMM - 2.0 - Services - DTU Health Tech) was used to predict the WT and mutant 5-HTR7A transmembrane domains. Transmembrane, intracellular, and extracellular domains are in red, blue, and purple, respectively.

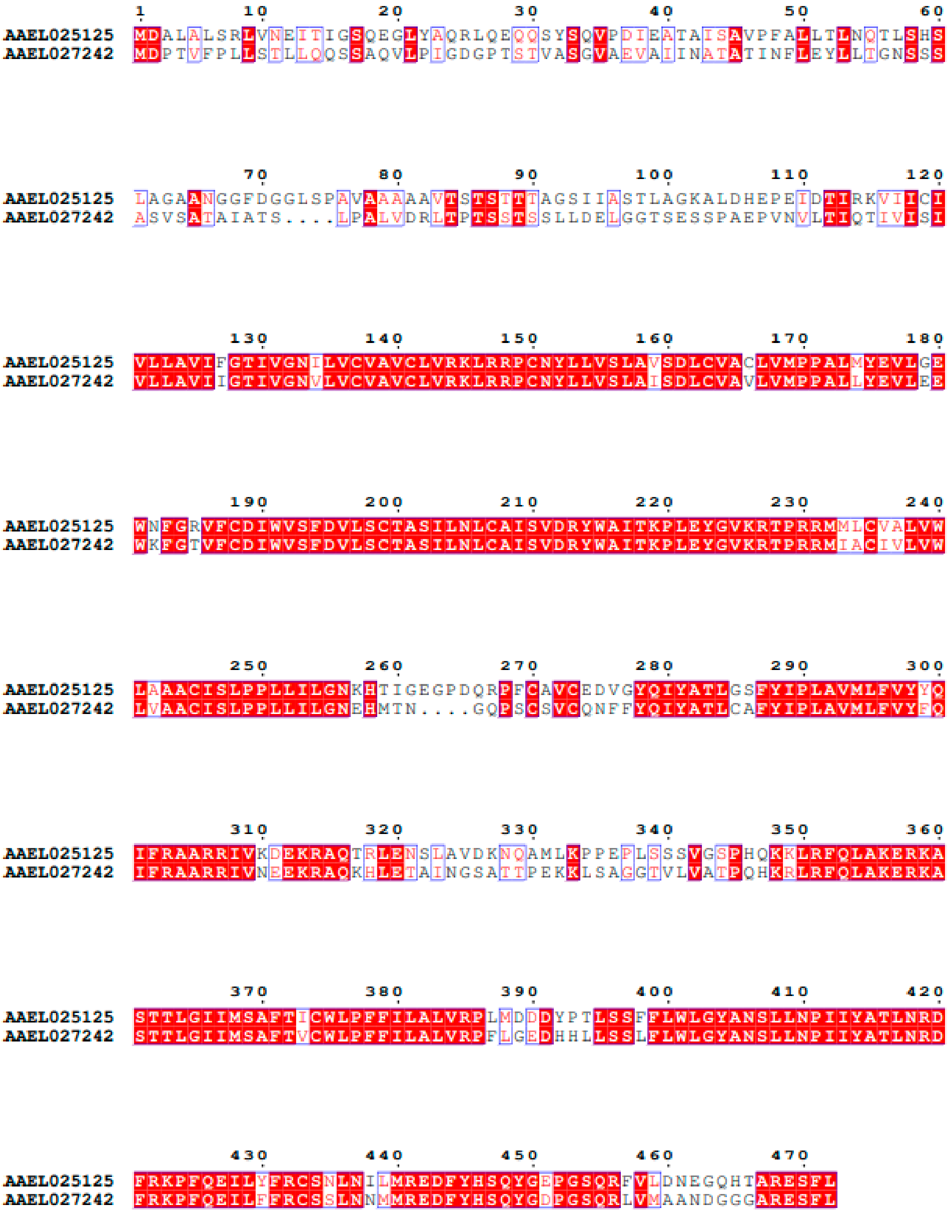

Figure S2. Sequence alignment between 5-HTR$_{7A}$ and 5-HTR$_{7B}$. 5-HTR$_{7A}$ (AAEL025125) and AAEL027242 (5-HTR$_{7B}$) were aligned using CLUSTALW ([Multiple Sequence Alignment - CLUSTALW (genome.jp)]). The results showed that the identity and similarity of two receptors were 61.44% and 86.23%, respectively.

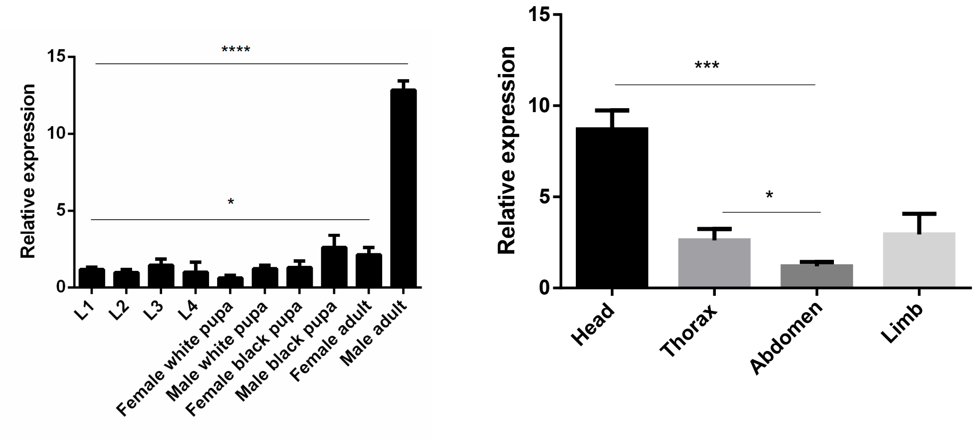

(**a**) (**b**)

Figure S3. *Ae. aegypti* 5-HTR$_{7B}$ temporal and spatial expression profiles. (a) *5-HTR*$_{7B}$ mRNA levels in 10 mosquito developmental stages, including eggs, larvae, and adults. (b) The differential expression levels of *5-HTR*$_{7B}$ receptor were analyzed in the head, thorax, abdomen, and limbs. Statistical analysis of the differential expression profiles was performed using T-tests (*P<0.05, **P<0.01, ***P<0.001, ****P<0.0001).

Supplementary tables

Table S1. Sequences of primers and sgRNA in this study

gene ID	Forward Primer (5'-3')	Reverse Primer (5'-3')
AAEL025999	AAGAAGTGGCCATCATTCCA	GGTCTCCGGGTCGACTTC
AAEL025125	ACGAAACCGCTAGAATACG	GTGGCAGCGATATACAGG
sgRNA	GAAATTAATACGACTCACTATAG GGTCCTTCTACATACCCCTGGGT TTTAGAGCTAGAAATAGC	AAAAGCACCGACTCGGTGCCACTTTTT CAAGTTGATAACGGACTAGCCTTATTT TAACTTGCTATTTCTAGCTCTAAAAC

Table S2. The proportion of major secondary protein structures of two 5-HTR proteins in *Ae.aegypti*.

Gene ID	α-helix(%)	Extended strand(%)	Beta turn(%)	Random coli(%)
AAEL025125	47.67	13.56	2.97	35.81
AAEL027242	47.64	17.67	3.23	31.47
